# Association of Academic Level and Body Mass Index with Depressive Symptoms Among Undergraduate and Graduate Students in Bronx, NY: A Cross-Sectional Study

**DOI:** 10.3390/bs16030400

**Published:** 2026-03-10

**Authors:** Aditi Puri, Peter C. Nwakeze, Collette M. Brown, Latoya Callender, Chesley Sanchez, William Suarez

**Affiliations:** 1 King Graduate School, Monroe University, Bronx, NY 104681, USA; pnwakeze@monroeu.edu; 2 School of Health Sciences, Human Services and Nursing, Lehman College, The City University of New York, New York, NY 10468, USA; collette.brown1@lehman.cuny.edu (C.M.B.); latoya.callender11@login.cuny.edu (L.C.);; 3 Department of Epidemiology and Biostatistics, School of Public Health, SUNY Downstate Health Science University, Brooklyn, NY 11203, USA; chesley.sanchez@downstate.edu

**Keywords:** academic level, body mass index, depression, obesity, overweight, sophomore

## Abstract

Depression and obesity are a public health crisis in the United States. A plethora of research has established an association between obesity and depression. Research on the relationship between normal weight, non-normal weight (underweight, overweight, and obesity), academic level, and depression among college students is limited. This study aims fills an important gap in the literature by analyzing the association between Body Mass Index (BMI) and depression by academic level. In addition, the interaction between BMI and depression by academic level was also evaluated. Data for this cross-sectional study were collected using a subscale (depression) of the Depression, Anxiety and Stress Scale (DASS-21) from 987 undergraduate and graduate students from two colleges in the Bronx, NY. BMI was calculated using participant’s self-reported height and weight. Data were analyzed using chi-square and logistic regression analyses. Results indicate that students in the normal weight category were less likely to be depressed compared to underweight, overweight, and obese students (Model 1: Adjusted Exp(B) = 0.641, C.I. = 0.416–0.989, and *p* = 0.044). Those who were in the freshman year were twice as likely to be depressed compared to graduate students (Model 1: Adjusted Exp(B) = 2.236, C.I. = 1.158–4.318, and *p* = 0.017). A significant interaction between BMI and academic level was found (Model 2: Adjusted Ex(B) = 5.404, C.I. = 1.114–26.221, and *p* = 0.036). This implies that the association between BMI and depression varies by academic level (sophomore). In conclusion institutions should develop programs that address risk factors for underweight, overweight, obesity, and depression simultaneously, with the goal of improving overall well-being and academic outcomes, especially among lower level (freshmen and sophomore) students.

## 1. Introduction

Obesity is commonly defined as having excessive body fat (Center of Disease Control, 2024). A benchmark for obesity is a Body Mass Index (BMI) of over 30 (Center of Disease Control, 2024). BMI is a screening tool used for estimating body fat using weight and height measurements (Cleveland Clinic, 2022). BMI is calculated as a person’s weight in kg divided by their height in meter^2^ (Center of Disease Control, 2024). Obesity is a complex and chronic disease that impacts overall health and quality of life. It is influenced by socioeconomic, environmental, medical, behavioral, and genetic factors such as excessive food intake, certain medications, disability, genetics, lack of sleep, stress, lack of physical activity, and underlying health issues such as metabolic syndrome (Cleveland Clinic, 2024). Obesity increases the risk of diseases and conditions such as cancer (esophageal, uterine, breast, pancreatic, and colorectal), female infertility, issues with memory/cognition, Alzheimer’s, dementia, depression and mood disorders, diabetes, high blood pressure, and joint problems (Cleveland Clinic, 2024; Center of Disease Control, 2022). The prevalence of obesity among adults in the United States between 2021 and 2023 was 40.3%, with no significant difference between men (39.2%) and women (41.3%) (Emmerich et al., 2024). In 2024, 28.7% of US college students described themselves as being overweight, while 6.6% considered themselves very obese (Elfin, 2025). The prevalence of obesity among American college students in 2024 was 18%, while 24% were overweight, and 5% were underweight (American College Health Association, 2024). Additionally, the prevalence of being slightly underweight (10%) and very underweight (1.1%) is much lower among college students in the US (Stewart, 2025).

Depression, also called a major depressive disorder (MDD), is a mood disorder that causes persistent feelings of sadness and lack of interest (Mayo Clinic, 2022). It is a serious disorder that negatively impacts how one thinks, acts, and feels. It is also called a major depressive disorder that results in varied emotional and physical problems (Mayo Clinic, 2022). According to the National Institutes of Health (National Institute of Health [NIH], 2023), approximately 21 million (8.3%) adults in the US had at least one major depressive episode in 2021. The NIH also indicated that the highest prevalence of major depressive episodes was highest among adults aged 18–25 years old (18.6%) and those who indicated more than one race (13.9%). College students are exposed to multiple risk factors including biological, college experience, their psychological and personality state, and lifestyle (Y. Zhang et al., 2023; Pedrelli et al., 2015) that result in depression and suicide. Specifically, the college experience varies by academic level; X. Wang et al. (2020) and X. Liu et al. (2019) found higher levels of depression among students in lower academic years. X. Liu et al. (2019) reported the highest level of depression among first- or second-year full-time undergraduate students. Similarly, X. Wang et al. (2020) also noted that students in higher academic levels (seniors and graduate students) reported lower depression scores compared to students in lower academic years (freshmen, sophomore, and junior). While depression rates among college students seem to be declining over the last three years, data from the 2024–2025 Healthy Minds Study (HMS) reveal that 37% of college students had Moderate to Severe depression, 17% were severely and 19% were moderately depressed, with only 37% of them receiving therapy (Eisenberg et al., 2025). Thus, mental health problems among college students are a growing public health challenge (Adams et al., 2021; Andrews & Wilding, 2004; Beiter et al., 2015; Busch et al., 2024; Charles et al., 2021; Dessauvagie et al., 2022; Duffy et al., 2019a, 2019b; Eisenberg et al., 2025; Li et al., 2023; X. Q. Liu et al., 2022; Luo et al., 2024; Pedrelli et al., 2015; Sheldon et al., 2021; SenthilKumar et al., 2023; X. Wang et al., 2020; Y. Zhang et al., 2023) that require immediate attention.

Previous studies have demonstrated conflicting evidence regarding the association between BMI and depression. Research illustrates that college students are often simultaneously impacted by obesity and depression (Akinyemi et al., 2022; Badillo et al., 2022; Barr-Porter et al., 2025; Cui et al., 2024; Cho et al., 2022; Darimont et al., 2020; de Wit et al., 2022; Hamurcu, 2023; Herhaus et al., 2020; Hossain et al., 2022; Joma et al., 2024; Kaufman et al., 2020; Luppino et al., 2010; Mannan et al., 2016; Nigatu et al., 2015; Odlaug et al., 2015; Pereira-Miranda et al., 2017; Sarigiani et al., 2020; Yu et al., 2011; H. Wang, 2024; Z. Wang et al., 2022; J. Zhang, 2021; Zhuang et al., 2025). Collectively, these studies indicate a positive relationship between BMI and depression, where higher levels of BMI correspond to higher levels of depression. Conversely, other studies report an association between being underweight and depression (Blasco et al., 2020; Carey et al., 2014; Cui et al., 2024; Jung et al., 2017; Khan et al., 2022; Xu et al., 2015). Cui et al. (2024) and Jung et al. (2017) noted that being underweight increased the risk of depression. In addition, a reciprocal relationship between depression and obesity exists, confirmed by Luppino et al. (2010) and Tzenios et al. (2023). Despite these correlations between BMI and depression, few studies found no significant association between the two variables (Aydemir, 2020; Pleșea-Condratovici et al., 2025; Zhou et al., 2020).

College campuses are adversely impacted by the increasing prevalence of obesity and depression. The literature establishes that those who are obese often suffer from depression (Barr-Porter et al., 2025; Hossain et al., 2022; Joma et al., 2024; Sethi et al., 2015). Moreover, mental health issues have a propensity to impact student success (Beiter et al., 2015). To our knowledge no studies evaluated the interaction between BMI and depression by academic level. Thus, our study fills an important gap in the literature by investigating the association between BMI and depression among students at various academic levels.

Purpose: This study was conducted among college students in the Bronx, NY, with the purpose of analyzing the association between Body Mass Index (BMI) and depression by academic level.

## 2. Materials and Methods

### 2.1. Study Design, Settings, and Participants

Students from a private university and public college located in the Bronx, NY, were recruited for this cross-sectional study. The student population was represented by undergraduate and graduate students. Nine hundred and eight-seven (987) students aged 18 years and older participated in this study. The Checklist for Reporting Results of Internet E-Surveys (CHERRIS) was used to report findings of an e-survey conducted for this study (Rampling et al., 2022).

Data were collected from students enrolled during the spring 2024 semester using Survey Monkey. Written approval was obtained from professors before approaching students in their classes. To recruit students from in-person and virtual classes, researchers described the study purpose, inclusion criteria, and consent procedure. Students who matched the inclusion criteria were given access to a QR code that led them to the survey. Students reviewed the informed consent before proceeding to answer the survey questions. They were not compensated for participation. The survey completion rate was 100%. It is important to note that Survey Monkey does not allow duplicate responses. The anonymous data was stored in a password protected computer with access limited to study researchers. In addition, strengthening the reporting of observational studies in epidemiology (STROBE) checklist was used to report the study findings (von Elm et al., 2007).

### 2.2. Measures

#### 2.2.1. Depression, Anxiety and Stress Scale (DASS) 21

The depression section of the DASS-21 scale was used to collect data from eligible participants. DASS-21 is a widely used screening measure used for assessing symptoms of depression, anxiety, and stress in community settings (Lovibond & Lovibond, 1995). The depression scale measures low self-esteem, low-positive effect, and hopelessness. The Cronbach’s alpha for Dass 21-D subscale was 0.72. The overall score, which includes all items, was also high (Cronbach’s alpha = 0.88). A few statements from the depression scale were I couldn’t seem to experience any positive feelings at all, I felt that life was meaningless, and I felt downhearted and blue. The scoring of the depression scale ranged from 0 to 42—Normal Depression (0–9), Mild Depression (10–12), Moderate (13–20), Severe (21–27), and Extremely Severe (28–42) (Lovibond & Lovibond, 1995; Tran et al., 2013). Self-reported height and weight can introduce measurement error. Moya et al. (2022) found a statistically significant association between DASS-21 depression subscale (DASS-D) and Edinburg Postnatal Depression Scale (EPDS) scores (r = 0.61, *p* < 0.001), indicating that DASS-21 is a reliable instrument for measuring both major and minor depression.

We calculated the sum of all the depression items (3, 5, 10, 13, 16, 17, and 21) to create a raw depression score. Because DASS21 is a shortened version of DASS42, all subscale totals must be multiplied by 2 to make them comparable to the original scale. The final depression variable was coded as follows: Normal: 0–9; Mild: 10–13; Moderate: 14–20; Severe: 21–27; and Extremely Severe: 28+.

#### 2.2.2. Body Mass Index

The Body Mass Index was calculated using participant’s self-reported height and weight. According to the Center of Disease Control (2024), BMI is calculated by dividing a person’s body weight (in kilograms) by the square of their height (in meters). The BMI categories are underweight (<18.5), healthy weight (18.5–24.9), overweight (25–29.9), and obese (≥30).

#### 2.2.3. Ethical Considerations

Institutional Review Board (IRB) approval was attained from both colleges in the Bronx, NY, USA (Monroe University (IRB No: FAC-2023-04), and Lehman College (IRB No. 2024-0087). All participants who met the eligibility criteria read the informed consent, responding “yes” to the question “Do you want to participate in the study?” After consenting, they proceeded to answer the survey questions. The study data were stored on the researchers’ password protected laptops.

#### 2.2.4. Data Analysis

Data were analyzed using Statistical Package for the Social Sciences (SPSS), version 29 statistical software (IBM Corp, 2025). Significant variables from the bivariate analysis were entered into a logistic regression model (Model 1) to control for potential confounders. In addition, an interaction term between BMI and academic levels (BMI × Freshmen, BMI × Sophomore, BMI × Junior, and BMI × Senior) was created and entered in the regression model (Model 2).

Bivariate analyses were conducted to identify candidate variables for inclusion in the multivariate logistic regression model. Of the variables targeted for evaluation (age, race, income, gender, degree level, GPA, and BMI), all except race were examined in relation to depression using chi-square tests. Race was excluded due to its highly uneven distribution across categories. Several racial groups had very small proportions: American Indian/Alaska Native (2.8%), Native Hawaiian (0.5%), and White non-Hispanic (6.5%). These distributions make it statistically inappropriate to collapse the variable into a minority versus non-minority dichotomy, particularly given that the non-minority group (white) represents only 6.5% of the sample.

Sensitivity analyses were conducted by modeling depression as a continuous DASS-21 score using multivariable linear regression and treating BMI/weight as continuous variables. These analyses used the same covariates as the primary logistic model. Although the direction of the association was consistent across models, the *p*-value in the continuous model was borderline, suggesting that the effect is modest.

Following the recommendation by (Kleinbaum & Klein, 2010), variables with a bivariate *p*-value ≤ 0.10 were retained as candidates for the multivariable model.

## 3. Results

### 3.1. Demographic Variables and Depression

Data were collected from 986 students from a private university and public college located in the Bronx, NY, USA. Table 1 shows the frequency distribution of demographic variables and depression. A majority of the students were female (70.2%) and aged 18–24 (56.5%). Minorities were highly represented in this sample, with 38.2% of the students being Black/African American, followed by Hispanics (32.2%). Most of the students (32.3%) had an annual family income below $20,000, while 26% had an income between $20,000–$40,000 and 16.9% between $40,001 and $60,000. A majority of the students were in their first year (29.3%), followed by graduate (26.6%), fourth year (15%), and third year (13.8%). A majority of the students were undergraduates (73%), and only 27% were graduates.

Approximately 51.3% of the students were either overweight (26.2%) or obese (25.1%). The prevalence of Severe (6.4%) and Extremely Severe (8.3%) depression was 14.7%, while 16.3% of the population experienced Moderate levels of depression. It is important to note that a majority of the students presented with Normal levels of depression (60.8%).

### 3.2. Bivariate Association Between Demographic Variable, BMI, and Depression (Crude Analysis)

Table 2 depicts the bivariate association between demographic variables, BMI vs. Depression. The variables included in the regession model were those that were significant (0.10 or less) at the bivariate level (age, gender, academic level, GPA, and BMI). We also created an interaction term between BMI and academic levels (BMI × AS Freshmen, BMI × AS Sophomore, BMI × BS Junior, and BMI × BS Senior).

### 3.3. Model 1: Logistic Regression Model (Adjusted): Factors Effecting Depression

A logistic regression model (Table 3) was used to predict the association between academic level, gender, BMI, and depression after controlling for potential confounders. The model predicts (predictors: age, gender, GPA, BMI, and academic level) 3% (Cox & Snell R) to 5% (Nagelkerke R square) variance in the outcome variable (depression). Students in the normal weight range were less likely to be depressed compared to those who were either obese, overweight, or underweight (Adjusted Exp(B) = −0.641, C.I. = 0.416–0.989, and *p* = 0.044). Those who were in their freshman year were twice as likely to be depressed compared to graduate students (Adjusted Exp(B) = 2.236, C.I. = 1.158–4.318, and *p* = 0.017); it is important to note that 12.5% of first-year students were very severely depressed and 6% were Severely depressed. In addition, men were less likely to be depressed compared to female students (Adjusted Exp(B) = 0.539, C.I. = 0.322–0.894, and *p* = 0.017).

### 3.4. Model 2: Logistic Regression Model (Adjusted): Interaction of Academic Level with BMI and Depression

The result shows that a normal weight was significantly associated with lower odds of depression among graduate students (Table 4–Model 1) but dropped in significance with the introduction of the interaction term for academic level and normal weight in Model 2. This points to a statistically significant interaction between BMI and academic level (Adjusted Ex(B) = 5.404, C.I. = 1.114–26.221, and *p* = 0.036). In other words, the analysis shows that the association between BMI and depression varies by academic level (sophomore). In conclusion, depression was higher among those who were sophomores compared to graduate students.

## 4. Discussion

### 4.1. BMI, Academic Level, and Depression

Our study found an association between BMI and depression, where those who were a normal weight were less likely to experience depressive symptoms compared to other categories of BMI. (Model 1: Adjusted Exp(B) = 0.641, C.I. = 0.416–0.989, and *p* = 0.044). Thus, any weight category (underweight, overweight, and obese) other than normal weight increased the risk of depression. In the regression model, we also found a significant association between being a freshman and depression (Model 1: Adjusted Exp(B) = 2.236, C.I. = 1.158–4.318, and *p* = 0.017). In our study, freshmen were twice as likely to experience depression compared to graduate students. Students in the freshmen year experienced the highest levels of Severe and Extremely Severe depression (18.5%), followed closely by sophomores (17%). Our study results lend support to findings by Ebert et al. (2019), X. Liu et al. (2019), and X. Wang et al. (2020), who found higher levels of depression among lower academic years.

However, when we conducted an interaction analysis, this relationship was no longer significant. We found a statistically significant interaction between BMI and academic level (Model 2: Adjusted Ex(B) = 5.404, C.I. = 1.114–26.221, and *p* = 0.036). The association between BMI and depression varied by academic level (sophomore). Depression was higher among those who were sophomores compared to graduate students. To our knowledge, no other study has evaluated the interaction between BMI and depression by academic level.

The association between underweight and depression is also noted in the literature (Carey et al., 2014; Cui et al., 2024; Jung et al., 2017; H. Wang, 2024). Cui et al. (2024) found that both obesity and being underweight increased the risk of depression among young, single, and highly educated participants. A systematic review of 183 studies also found that the risk of depression increased among those who were underweight in both cross-sectional and longitudinal studies (Jung et al., 2017). H. Wang (2024) found that both BMI (*p* = 0.006) and being underweight (*p* = 0.03) were associate with depression.

### 4.2. Implications

This study had three major findings: the prevalence of Mild (8.2%), Moderate (16.3%), Severe–Extremely Severe (14.7%) depression (39.2%), being overweight (26.2%), and obesity (25.1%) were high among this population. Normal weight was associated with depression (*p* = 0.044). In addition, the effect of BMI on depression varied by academic level (sophomore) (*p* = 0.036); this finding fills an important gap in the literature, as no other study to our knowledge has studied this interaction.

The prevalence of depression in our study (39%) was almost similar to depression levels among college students in the US, as noted by the Healthy Minds study (37%) (Eisenberg et al., 2025). However, the prevalence of obesity (51%) was much higher among our population compared to the obesity levels in the US (40.3%) (Emmerich et al., 2024). The borough of the Bronx has the highest prevalence of obesity (34%) compared to Manhattan (17.2%) (New York State Department of Health, 2023). Moreover, the elevated prevalence of depression in this sample may reflect the environmental stressors associated with the Bronx, such as food insecurity, neighborhood disadvantage, and housing instability (Brown et al., 2025; Sanborn et al., 2025). Integrated therapies that target both obesity and depression can address both crises simultaneously. Ma et al. (2019) recommend integrating problem-solving therapy for depression with Diabetes Prevention Program-based behavioral weight loss.

A significant finding of our study was that the interaction between BMI and depression varied by academic level (sophomore). In other words, sophomores exhibited higher levels of depression compared to graduate students. Thus, social and emotional support should be provided to sophomores or students in lower academic years to address the dual burden of obesity and depression. Colleges should also invest in support services such as mental health counseling, food pantries, fruit and vegetable vouchers, physical activity initiatives, health and wellness programs, housing assistance, and child care (Society for Public Health Education [SOPHE], 2025). Food and Drug Association (FDA)-approved drugs such as NB-ER Phentermine-Topiramates-ER, GLP-1R agonist, Liraglutide Semaglutide, and Tirzepatide should be used for obesity treatment in addition to lifestyle modification (Chen et al., 2024; Kushner et al., 2025). In addition, public health initiatives such as the Bronx Health Reach can be implemented to address obesity on college campuses.

Universities can rely on faculty as first responders to identify students in mental health distress (Abrams, 2022). An academic environment that promotes physical and mental well-being is critical for the overall success of students, especially students in lower academic years (SenthilKumar et al., 2023). Most importantly, academic institutions in the Bronx should target mental and physical health interventions towards students in their sophomore year. In addition, further research is required to understand why sophomores have higher levels of depression compared to graduate students.

## 5. Limitations

Several limitations impact this study. A notable limitation is the use of a cross-sectional design. While this design is effective for identifying associations and prevalences at a single point in time, it cannot establish causality. Thus, we are unable to establish a causal relationship between BMI and depression. One of the potential limitations as a survey collected via “Survey Monkey” is that potential and unnoticed technical issues and attrition bias could happen. For instance, during the process, the participants were using different devices (mobile phones, tablets, laptops, etc.) that might bring display issues. In addition, the number of screens or items per page, font size, and type on page might be limited by the type of device used by the participant, which may discourage participation (Rampling et al., 2022). Participants were recruited by researchers from specific undergraduate and graduate classes. This non-probability sampling method may lead to selection bias, as students who chose to participate might differ significantly in their current mental state or overall health from those who did not. The research relied exclusively on subjective, self-reported measures for the key variables. Self-reported health outcomes are vulnerable to systematic selection and information biases, potentially undermining the validity. A systematic review by Fayyaz et al.’s (2024) found an agreement between self-reported and measured height and weight. The measured weight and height have a higher validity and reliability. However, self-reported measures are a valuable supplement for direct anthropometric data.

The bias is not uniform across subgroups, as individuals with elevated depressive symptoms underreport weight compared with those with lower depression. Thus, the observed odds ratio of OR = 0.641 in this study may represent a lower estimate of the true association between BMI and depression.

This study’s findings cannot be generalized beyond the college student population in the Bronx, NY. It is important to note that the Bronx has the highest rates of obesity in NYC at 37.4% compared to 20.2% in Manhattan (NYC.gov, 2022). In our study, the prevalence of obesity (51%) was even higher. Moreover, 26.5% of households live below the poverty line in the Bronx compared to 17% of households in NYC. Such households are deprived of resources such as good quality housing, healthcare, and other means that protect health (NYC.gov, 2022).

## 6. Conclusions

The contribution of our study to public health is significant, as it explored an important association between BMI, academic level, and depression. We found that academic level and BMI were significantly associated with depression among college students. To our knowledge, no study has explored an interaction between BMI and academic level. Our study shows that the association between BMI and depression varies by academic level (sophomore). Colleges should invest in programs and policies that concurrently address obesity and depression among students. The interventions should be specifically targeted towards students in lower academic years (freshmen and sophomores). In fact, sophomores presented with higher levels of depression compared to graduate students. Therefore, mental health and weight management interventions should be tailored to meet the needs of sophomores. Most importantly colleges should create an academic environment that promotes health and mental well-being among students in the lower academic years.

## Figures and Tables

**Table 1:** Frequency distribution of demographic variables and depression.

Variable	Number (*n*)	Percent (%)
**Age** (*n* = 986)		
18–24	557	56.5
25–34	294	29.5
35–44	97	10.0
45+	38	4.0
**Race** (*n* = 978)		
American Indian/Alaska Natives—Non-Hispanic	27	2.8
Asian—Non-Hispanic	194	19.8
Black or African American, Non-Hispanic	374	38.2
Native Hawaiian/Pacific Islander—Non-Hispanic	5	0.5
Hispanic	314	32.2
White—Non-Hispanic	64	6.5
**Income** (*n* = 954)		
<$20,000	306	32.3
$20,000–$40,000	244	26.0
$40,001–$60,000	161	16.9
$60,001–$80,000	113	11.8
$800,001+	130	13.0
**Gender** (*n* = 987)		
Male	287	29.1
Female	693	70.2
Non-Binary	2	0.2
Other	5	0.5
**GPA** (*n* = 900)		
<3	179	19.9
3.0–3.49	255	28.3
3.5–4.0	466	11.9
**Degree Level** (*n* = 983)		
Freshman—1st year	288	29.3
Sophomore—2nd year	150	15.3
Junior—3rd Year	136	13.8
Senior—4th Year	148	15.0
Graduate School	261	26.6
**School Level** (*n* = 983)		
Undergraduate	722	73.0
Graduate	261	27.0
**BMI** (*n* = 924)		
Underweight	68	7.4
Normal	382	41.3
Overweight	242	26.2
Obese	232	25.1
**Depression** (*n* = 967)		
Normal	588	60.8
Mild	79	8.2
Moderate	158	16.3
Severe	62	6.4
Extremely Severe	80	8.3

**Table 2:** Bivariate association demographic variable, BMI, and depression (crude analysis).

Variable	Normal Depression *n* (%)	Mild Depression *n* (%)	Moderate Depression *n* (%)	Severe Depression *n* (%)	Extremely Severe Depression *n* (%)	Chi-Square	df	*p*-Value
**Age**								
18–24	298 (54.8)	42 (7.7)	112 (20.6)	39 (7.2)	53 (9.7)	33.411	12	<0.001 *
25–34	191 (66.3)	25 (8.7)	33 (11.5)	21 (7.3)	18 (6.3)			
35–44	70 (72.9)	8 (8.3)	11 (11.5)	0 (0)	7 (7.3)			
45+	28 (73.7)	4 (10.5)	2 (5.3)	2 (5.3)	2 (5.3)			
**Gender**								
Male	186 (67.4)	24 (8.7)	38 (13.8)	11 (4)	17 (6.2)	27.556	12	0.006 *
Female	400 (58.5)	55 (8)	119 (17.4)	49 (7.2)	61 (8.9)			
Non-Binary	0 (0)	0 (0)	1 (50)	0 (0)	1 (50)			
Other	2 (40)	0 (0)	0 (0)	2 (40)	1 (20)			
**Income**								
<$20,000	168 (56.8)	24 (8.1)	50 (16.9)	22 (7.4)	32 (10.8)	30.445	24	0.170
$20,000–$40,000	150 (62.2)	26 (10.8)	32 (13.3)	16 (6.6)	17 (7.1)			
$40,001–60,000	94 (59.1)	14 (8.8)	29 (18.2)	14 (8.8)	8 (5)			
$600,001–80,000	67 (60.4)	7 (6.3)	24 (21.6)	4 (3.6)	9 (8.1)			
80,001–100,000	50 (74.6)	6 (9)	3 (4.5)	3 (4.5)	5 (7.5)			
1,000,001–120,000	21 (70)	1 (3.3)	6 (20)	1 (3.3)	1 (3.3)			
120,000+	21 (65.6)	1 (3.1)	6 (18.8)	0 (0)	4 (12.5)			
**Academic Level**								
Freshmen	149 (53)	23 (8.2)	57 (20.3)	17 (6)	35 (12.5)	31.253	16	0.012 *
Sophomore	87 (59.2)	17 (11.6)	18 (12.2)	12 (8.2)	13 (8.8)			
Junior	86 (63.2)	12 (8.8)	21 (15.4)	11 (8.1)	6 (4.4)			
Senior	87 (59.2)	12 (8.2)	25 (17)	8 (5.4)	15 (10.2)			
Graduate	176 (70.4)	15 (6)	34 (13.6)	14 (5.6)	11 (4.4)			
**School Level**								
Undergraduate	409 (57.5)	64 (9)	121 (17)	48 (6.8)	69 (9.7)	15.036	4	0.005 *
Graduate	176 (70.4)	15 (6)	34 (13.6)	14 (5.6)	11 (4.4)			
**GPA**								
<3	91 (52)	17 (9.7)	34 (19.4)	13 (7.4)	20 (11.4)	17.081	8	0.029 *
3.0–3.49	146 (58.9)	27 (10.9)	37 (14.9)	14 (5.6)	24 (9.7)			
3.5–4.0	302 (65.4)	31 (6.7)	73 (15.8)	31 (6.7)	25 (5.4)			
**BMI**								
Underweight	44 (66.7)	5 (7.6)	8 (12.1)	3 (4.5)	6 (9.1)	10.331	12	0.587
Normal	247 (65.2)	26 (6.9)	59 (15.6)	22 (5.8)	25 (6.6)			
Overweight	130 (54.6)	25 (10.5)	43 (18.1)	17 (7.1)	23 (9.7)			
Obese	133 (58.1)	20 (8.7)	38 (16.6)	16 (7)	22 (9.6)			

* *p* value < 0.05.

**Table 3:** Model 1: logistic regression model 1: factors effecting depression.

Variable	B	SE	Wald	df	Sig	Exp(B)	95% C.I.	
**Age**							Lower	Upper
18–24	0.376	0.563	0.446	1	0.504	1.457	0.483	4.896
25–34	0.309	0.575	0.290	1	0.590	1.363	0.442	4.203
35–44	−0.329	0.671	0.241	1	0.624	0.719	0.193	2.681
**BMI**								
Normal Weight	−0.444	0.221	4.040	1	0.044 *	0.641	0.416	0.989
**Gender**								
Male	−0.623	0.261	5.708	1	0.017 *	0.536	0.322	0.894
**Academic Level**								
Freshmen	0.805	0.336	5.747	1	0.017 *	2.236	1.158	4.318
Sophomore	0.540	0.361	2.235	1	0.135	1.716	0.845	3.482
Junior	0.218	0.385	0.320	1	0.571	1.244	0.585	2.645
Senior	0.378	0.357	1.120	1	0.290	1.459	0.725	2.935
**GPA**								
<3.0	−0.199	0.243	0.670	1	0.413	0.820	0.509	1.320
Constant	−2.060	0.617	11.137	1	<0.001	0.127		

* *p* value < 0.05.

**Table 4:** Model 2: logistic regression model: interaction of academic level with BMI and depression.

Variable	B	SE	Wald	df	Sig	Exp(B)	95% C.I.	
**Age**							Lower	Upper
18–24	0.376	0.563	0.427	1	0.513	1.447	0.478	4.377
25–34	0.339	0.576	0.347	1	0.556	1.403	0.454	4.336
35–44	−0.346	0.672	0.266	1	0.606	0.707	0.189	2.640
**BMI**								
Normal Weight	−1.378	0.652	4.472	1	0.034 *	0.252	0.070	0.904
**Gender**								
Male	−0.623	0.261	5.708	1	0.017 *	0.536	0.322	0.894
**Academic Level**								
BMI × Freshmen	0.930	0.742	1.569	1	0.210	2.534	0.591	10.854
BMI × Sophomore	1.687	0.806	4.384	1	0.036 *	5.404	1.114	26.221
BMI × Junior	0.099	1.025	0.009	1	0.923	1.104	0.148	8.227
BMI × Senior	1.339	0.841	2.533	1	0.112	3.814	0.733	19.831
**GPA**								
<3.0	−0.213	0.244	0.804	1	0.370	0.803	0.498	1.296
Constant	−1.811	0.631	8.246	1	0.004	0.163		

* *p* value < 0.05.

## Data Availability

The data is available upon request from the corresponding author. Data is not available publicly due to privacy and ethical restrictions.

## Abbreviations

The following abbreviations are used in this manuscript:

BMI	Body Mass Index
DASS-21	Depression, Anxiety and Stress Scale

## References

[B1-behavsci-16-00400] Abrams Z. (2022). Student mental health is in crisis. Campuses are rethinking their approach.

[B2-behavsci-16-00400] Adams K. L., Saunders K. E., Keown-Stoneman C. D. G., Duffy A. C. (2021). Mental health trajectories in undergraduate students over the first year of university: A longitudinal cohort study. BMJ.

[B3-behavsci-16-00400] Akinyemi O. A., Babatunde O., Weldeslase T. A., Akinyemi I., Akinwumi B., Oladunjoye A. O., Ogundare T., Bezold M. (2022). Association between obesity and self-reported depression among female university students in the United States. Cureus.

[B4-behavsci-16-00400] American College Health Association (2024). National college health assessment III: Reference group executive summary.

[B5-behavsci-16-00400] Andrews B., Wilding J. M. (2004). The relation of depression and anxiety to life-stress and achievement in students. British Journal of Psychology (London, England: 1953).

[B6-behavsci-16-00400] Aydemir İ. (2020). Examining the level of depression among university students and evaluating its relationship with body mass index (BMI). Clinical and Experimental Health Sciences.

[B7-behavsci-16-00400] Badillo N., Khatib M., Kahar P., Khanna D. (2022). Correlation between body mass index and depression/depression-like symptoms among different genders and races. Cureus.

[B8-behavsci-16-00400] Barr-Porter M., Sullivan A., McNamara J. (2025). Body mass index, sleep, and food insecurity predict prevalence of anxiety and depression in college students. American Journal of Lifestyle Medicine.

[B9-behavsci-16-00400] Beiter R., Nash R., McCrady M., Rhoades D., Linscomb M., Clarahan M., Sammut S. (2015). The prevalence and correlates of depression, anxiety, and stress in a sample of college students. Journal of Affective Disorders.

[B10-behavsci-16-00400] Blasco B. V., García-Jiménez J., Bodoano I., Gutiérrez-Rojas L. (2020). Obesity and depression: Its prevalence and influence as a prognostic factor: A systematic review. Psychiatry Investigation.

[B11-behavsci-16-00400] Brown C. M., Nwakeze P. C., Puri A., Sanchez C., Callender L., Williams E. V., Suarez W. (2025). Association between food insecurity and mental health among college students in the Bronx, New York (NY). Nutrients.

[B12-behavsci-16-00400] Busch C. A., Wiesenthal N. J., Gin L. E., Cooper K. M. (2024). Behind the graduate mental health crisis in science. Nature Biotechnology.

[B13-behavsci-16-00400] Carey M., Small H., Yoong S. L., Boyes A., Bisquera A., Sanson-Fisher R. (2014). Prevalence of comorbid depression and obesity in general practice: A cross-sectional survey. The British Journal of General Practice: The Journal of the Royal College of General Practitioners.

[B14-behavsci-16-00400] Center of Disease Control (2022). Overweight and obesity.

[B15-behavsci-16-00400] Center of Disease Control (2024). Adult BMI categories.

[B16-behavsci-16-00400] Charles S. T., Karnaze M. M., Leslie F. M. (2021). Positive factors related to graduate student mental health. Journal of American College Health.

[B17-behavsci-16-00400] Chen X., Zhao P., Wang W., Guo L., Pan Q. (2024). The antidepressant effects of GLP-1 receptor agonists: A systematic review and meta-analysis. The American Journal of Geriatric Psychiatry: Official Journal of the American Association for Geriatric Psychiatry.

[B18-behavsci-16-00400] Cho Y. K., Lee Y., Jung C. H. (2022). Pathogenesis, murine models, and clinical implications of metabolically healthy obesity. International Journal of Molecular Sciences.

[B19-behavsci-16-00400] Cleveland Clinic (2022). Body mass index.

[B20-behavsci-16-00400] Cleveland Clinic (2024). Obesity.

[B21-behavsci-16-00400] Cui H., Xiong Y., Wang C., Ye J., Zhao W. (2024). The relationship between BMI and depression: A cross-sectional study. Frontiers in Psychiatry.

[B22-behavsci-16-00400] Darimont T., Karavasiloglou N., Hysaj O., Richard A., Rohrmann S. (2020). Body weight and self-perception are associated with depression: Results from the national health and nutrition examination survey (NHANES) 2005–2016. Journal of Affective Disorders.

[B23-behavsci-16-00400] Dessauvagie A. S., Dang H. M., Nguyen T. A. T., Groen G. (2022). Mental health of university students in Southeastern Asia: A systematic review. Asia-Pacific Journal of Public Health.

[B24-behavsci-16-00400] de Wit L., Have M. T., Cuijpers P., de Graaf R. (2022). Body mass index and risk for onset of mood and anxiety disorders in the general population: Results from the Netherlands mental health survey and incidence study-2 (NEMESIS-2). BMC Psychiatry.

[B25-behavsci-16-00400] Duffy A., Keown-Stoneman C., Goodday S., Horrocks J., Lowe M., King N., Pickett W., McNevin S. H., Cunningham S., Rivera D., Bisdounis L., Bowies C. R., Harkness K., Saunders K. E. A. (2019a). Predictors of mental health and academic outcomes in the first-year university students: Identifying prevention and early intervention targets. BJPsych Open.

[B26-behavsci-16-00400] Duffy A., Saunders K. E. A., Malhi G. S., Patten S., Cipriani A., McNevin S. H., MacDonald E., Geddes J. (2019b). Mental health care for university students: A way forward?. The Lancet: Psychiatry.

[B27-behavsci-16-00400] Ebert D. D., Buntrock C., Mortier P., Auerbach R., Weisel K. K., Kessler R. C., Cuijpers P., Green J. G., Kiekens G., Nock M. K., Demyttenaere K., Bruffaerts R. (2019). Prediction of major depressive disorder onset in college students. Depression and Anxiety.

[B28-behavsci-16-00400] Eisenberg D., Lipson S. K., Heinze J., Zhou S. (2025). The healthy minds study.

[B29-behavsci-16-00400] Elfin J. (2025). How US college students describe their weight as of fall 2024.

[B30-behavsci-16-00400] Emmerich S. D., Fryar C. D., Stierman B., Ogden C. L. (2024). Obesity and severe obesity prevalence in adults: United States, August 2021–August 2023.

[B31-behavsci-16-00400] Fayyaz K., Bataineh M. F., Ali H. I., Al-Nawaiseh A. M., Al-Rifai’ R. H., Shahbaz H. M. (2024). Validity of measured vs. self-reported weight and height and practical considerations for enhancing reliability in clinical and epidemiological studies: A systematic review. Nutrients.

[B32-behavsci-16-00400] Hamurcu P. (2023). Impact of perceived body weight on depression, anxiety and stress levels of young adults in Turkey. Iranian Journal of Public Health.

[B33-behavsci-16-00400] Herhaus B., Kersting A., Brähler E., Petrowski K. (2020). Depression, anxiety and health status across different BMI classes: A representative study in Germany. Journal of Affective Disorders.

[B34-behavsci-16-00400] Hossain A., Bhuiya R. A., Ali M. Z. (2022). The association between obesity and depression, anxiety, and stress disorders among university students at Rajshahi City in Bangladesh. Journal of Psychiatry and Psychiatric Disorders.

[B35-behavsci-16-00400] IBM Corp (2025). IBM SPSS statistics for windows *(Version 29.0) [Computer software]*.

[B36-behavsci-16-00400] Joma A., Abuzerr S., Alsoudi S. (2024). Variables associated with the relationship between obesity and mental health among university students in the Gulf Cooperation Council countries: A systematic review. Frontiers in Public Health.

[B37-behavsci-16-00400] Jung S. J., Woo H. T., Cho S., Park K., Jeong S., Lee Y. J., Kang D., Shin A. (2017). Association between body size, weight change and depression: Systematic review and meta-analysis. The British Journal of Psychiatry: The Journal of Mental Science.

[B38-behavsci-16-00400] Kaufman C. C., Thurston I. B., Maclin-Akinyemi C., Hardin R. N., Decker K. M., Kamody R. C. (2020). Risk and protective factors associated with depressive symptoms in young adults with overweight and obesity. Journal of American College Health.

[B39-behavsci-16-00400] Khan B. A., Khalid H., Siddiqi N., Aslam F., Ayesha R., Afzal M., Rajan S., Appuhamy K., Nahar Koly K., Bryant M., Holt R. I. G., Zavala Gomez G. A. (2022). Prevalence of underweight in people with severe mental illness: Systematic review and meta-analysis. Mental Health Science.

[B40-behavsci-16-00400] Kleinbaum D. G., Klein M. (2010). Logistic regression: A self-learning text.

[B41-behavsci-16-00400] Kushner P., Kahan S., McIntyre R. S. (2025). Treating obesity in patients with depression: A narrative review and treatment recommendation. Postgraduate Medicine.

[B42-behavsci-16-00400] Li L., Zhu M., Yao A., Yang J., Yang L. (2023). Daily stress, and mental health of professional degree graduate students in Chinese traditional medicine universities: The mediating role of learning career adaptation. BMC Medical Education.

[B43-behavsci-16-00400] Liu X., Ping S., Gao W. (2019). Changes in undergraduate students’ psychological well-being as they experience university life. International Journal of Environmental Research and Public Health.

[B44-behavsci-16-00400] Liu X. Q., Guo Y. X., Zhang W. J., Gao W. J. (2022). Influencing factors, prediction and prevention of depression in college students: A literature review. World Journal of Psychiatry.

[B45-behavsci-16-00400] Lovibond S. H., Lovibond P. F. (1995). Manual for the depression anxiety stress scales.

[B46-behavsci-16-00400] Luo M. M., Hao M., Li X. H., Liao J., Wu C. M., Wang Q. (2024). Prevalence of depressive tendencies among college students and the influence of attributional styles on depressive tendencies in the post-pandemic era. Frontiers in Public Health.

[B47-behavsci-16-00400] Luppino F. S., de Wit L. M., Bouvy P. F., Stijnen T., Cuijpers P., Penninx B. W., Zitman F. G. (2010). Overweight, obesity, and depression: A systematic review and meta-analysis of longitudinal studies. Archives of General Psychiatry.

[B48-behavsci-16-00400] Ma J., Rosas L. G., Lv N., Xiao L., Snowden M. B., Venditti E. M., Lewis M. A., Goldhaber-Fiebert J. D., Lavori P. W. (2019). Effect of integrated behavioral weight loss treatment and problem-solving therapy on body mass index and depressive symptoms among patients with obesity and depression: The RAINBOW randomized clinical trial. JAMA.

[B49-behavsci-16-00400] Mannan M., Mamun A., Doi S., Clavarino A. (2016). Is there a bi-directional relationship between depression and obesity among adult men and women? Systematic review and bias-adjusted meta analysis. Asian Journal of Psychiatry.

[B50-behavsci-16-00400] Mayo Clinic (2022). Depression (major depressive disorder).

[B51-behavsci-16-00400] Moya E., Larson L. M., Stewart R. C., Fisher J., Mwangi M. N., Phiri K. S. (2022). Reliability and validity of depression anxiety stress scale (DASS)-21 in screening for common mental disorders among postpartum women in Malawi. BMC Psychiatry.

[B52-behavsci-16-00400] National Institute of Health [NIH] (2023). Major depression.

[B53-behavsci-16-00400] New York State Department of Health (2023). Self-reported food insecurity among New York State adults by county, BRFSS 20121.

[B54-behavsci-16-00400] Nigatu Y. T., Bültmann U., Reijneveld S. A. (2015). The prospective association between obesity and major depression in the general population: Does single or recurrent episode matter?. BMC Public Health.

[B55-behavsci-16-00400] NYC.gov (2022). Environmental health data portal.

[B56-behavsci-16-00400] Odlaug L. B., Lust K., Wimmelmann L. C., Chamberlain S. R., Mortensen E. K., Derbyshire K., Christenson G., Grant J. E. (2015). Prevalence and correlates of being overweight and obese in college. Psychiatry Research.

[B57-behavsci-16-00400] Pedrelli P., Nyer M., Yeung A., Zulauf C., Wilens T. (2015). College students: Mental health problems and treatment considerations. Academic Psychiatry: The Journal of the American Association of Directors of Psychiatric Residency Training and the Association for Academic Psychiatry.

[B58-behavsci-16-00400] Pereira-Miranda E., Costa P. R. F., Queiroz V. A. O., Pereira-Santos M., Santana M. L. P. (2017). Overweight and obesity associated with higher depression prevalence in adults: A systematic review and meta-analysis. Journal of the American College of Nutrition.

[B59-behavsci-16-00400] Pleșea-Condratovici C., Dionisie V., Moroianu L. A., Serban P. S., Plesea-Condratovici V., Arbune M. (2025). Perception versus actual weight: Body image dissatisfaction as a stronger correlate of anxiety and depression than BMI among Romanian health sciences students. Healthcare.

[B60-behavsci-16-00400] Rampling C. M., Gupta C. C., Shriane A. E., Ferguson S. A., Rigney G., Vincent G. E. (2022). Does knowledge of sleep hygiene recommendations match behaviour in Australian shift workers? A cross-sectional study. BMJ Open.

[B61-behavsci-16-00400] Sanborn J., Sisselman-Borgia A., Jones H. E., Twiste T., Freudenberg N. (2025). Combined effects of food and housing insecurity on academic attrition: Findings from a population-representative survey of urban public university students. Journal of American College Health.

[B62-behavsci-16-00400] Sarigiani P. A., Olsavsky A. L., Camarena P. M., Sullivan S. M. (2020). Obesity and depressive symptoms in college women: Analysis of body image experiences and comparison to non-obese women. International Journal of Adolescence and Youth.

[B63-behavsci-16-00400] SenthilKumar G., Mathieu N. M., Freed J. K., Sigmund C. D., Gutterman D. D. (2023). Addressing the decline in graduate students’ mental well-being. American Journal of Physiology-Heart and Circulatory Physiology.

[B64-behavsci-16-00400] Sethi S. R., Juneja R., Tripathi V. (2015). Obesity, gender and depression among college-going students. The International Journal of Humanities & Social Studies.

[B65-behavsci-16-00400] Sheldon E., Simmonds-Buckley M., Bone C., Mascarenhas T., Chan N., Wincott M., Gleeson H., Sow K., Hind D., Barkham M. (2021). Prevalence and risk factors for mental health problems in university undergraduate students: A systematic review with meta-analysis. Journal of Affective Disorders.

[B66-behavsci-16-00400] Society for Public Health Education (2025). Reach urban communities.

[B67-behavsci-16-00400] Stewart C. (2025). Percentage of college students that described themselves as underweight or overweight as of fall 2024.

[B68-behavsci-16-00400] Tran T. D., Tran T., Fisher J. (2013). Validation of the depression anxiety stress scales (DASS) 21 as a screening instrument for depression and anxiety in a rural community-based cohort of northern Vietnamese women. BMC Psychiatry.

[B69-behavsci-16-00400] Tzenios P. N., Tazanios M., Chahine M., Binti Jamal P. O. (2023). The complex relationship between obesity and depression. Special Journal of the Medical Academy and Other Life Sciences.

[B70-behavsci-16-00400] von Elm E., Altman D. G., Egger M., Pocock S. J., Gøtzsche P. C., Vandenbroucke J. P., STROBE Initiative (2007). Strengthening the reporting of observational studies in epidemiology (STROBE) statement: Guidelines for reporting observational studies. BMJ.

[B71-behavsci-16-00400] Wang H. (2024). Association between depression and BMI in young adults. Transactions on Materials, Biotechnology and Life Sciences.

[B72-behavsci-16-00400] Wang X., Hegde S., Son C., Keller B., Smith A., Sasangohar F. (2020). Investigating mental health of US college students during the COVID-19 pandemic: Cross-sectional survey study. Journal of Medical Internet Research.

[B73-behavsci-16-00400] Wang Z., Cheng Y., Li Y., Han J., Yuan Z., Li Q., Zhong F., Wu Y., Fan X., Bo T., Gao L. (2022). The relationship between obesity and depression is partly dependent on metabolic health status: A nationwide inpatient sample database study. Frontiers in Endocrinology.

[B74-behavsci-16-00400] Xu Y., Zhou Z., Li Y., Yang J., Guo X., Gao J., Yan J., Chen G. (2015). Exploring the nonlinear relationship between body mass index and health-related quality of life among adults: A cross-sectional study in Shaanxi province, China. Health and Quality of Life Outcomes.

[B75-behavsci-16-00400] Yu N. W., Chen C. Y., Liu C. Y., Chau Y. L., Chang C. M. (2011). Association of body mass index and depressive symptoms in a Chinese community population: Results from the health promotion knowledge, attitudes, and performance survey in Taiwan. Chang Gung Medical Journal.

[B76-behavsci-16-00400] Zhang J. (2021). The bidirectional relationship between body weight and depression across gender: A simultaneous equation approach. International Journal of Environmental Research and Public Health.

[B77-behavsci-16-00400] Zhang Y., Tao S., Qu Y., Mou X., Gan H., Zhou P., Zhu Z., Wu X., Tao F. (2023). The correlation between lifestyle health behaviors, coping style, and mental health during the COVID-19 pandemic among college students: Two rounds of a web-based study. Frontiers in Public Health.

[B78-behavsci-16-00400] Zhou Y., Yang G., Peng W., Zhang H., Peng Z., Ding N., Guo T., Cai Y., Deng Q., Chai X. (2020). Relationship between depression symptoms and different types of measures of obesity (BMI, SAD) in US women. Behavioural Neurology.

[B79-behavsci-16-00400] Zhuang H., Zhao H., Liu M., Wang Y., Wang Y., He C., Zhai J., Wang B. (2025). Depression, anxiety, stress symptoms among overweight and obesity in medical students, with mediating effects of academic burnout and internet addiction. Scientific Reports.

